# Focus on the Role of Non-Invasive Respiratory Support (NRS) during Palliative Care in Patients with Life-Limiting Respiratory Disease

**DOI:** 10.3390/jcm13175165

**Published:** 2024-08-30

**Authors:** Giorgia Spinazzola, Giuliano Ferrone, Teresa Michi, Flavia Torrini, Stefania Postorino, Fabio Sbaraglia, Loretta Gulmini, Massimo Antonelli, Giorgio Conti, Savino Spadaro

**Affiliations:** 1 Department of Anesthesia and Intensive Care, Fondazione Policlinico Universitario A. Gemelli IRCCS, 00168 Rome, Italy; giorgia.spinazzola@policlinicogemelli.it (G.S.);; 2 Palliative Care/Hospice, University of Ferrara, 44100 Ferrara, Italy; 3 Department of Translational Medicine, University of Ferrara, Azienda Ospedaliera-Universitaria di Ferrara, 44100 Ferrara, Italy; spdsvn@unife.it; 4 Department of Anesthesia and Intensive Care, Catholic University of the Sacred Heart, 00168 Rome, Italy

**Keywords:** palliative care, non-invasive respiratory support, respiratory failure, dyspnea, chronic respiratory disease

## Abstract

The management of patients with life-threatening respiratory disease in the ICU and at home has become increasingly of interest over the past decades. Growing knowledge supports the use of NRS, aimed at improving patient comfort and improving quality of life. However, its role during palliative care is not well defined, and evidence of support remains limited. The aim of this narrative review is to examine the recent evidence relating to the use of non-invasive respiratory support at the end of life, in order to clarify who benefits and when. The literature research was conducted on PubMed, using MeSH words. A review of the relevant literature showed that non-invasive respiratory support techniques for patients with life-limiting respiratory disease vary (from high-flow oxygen therapy to conventional oxygen therapy, from CPAP to NPPV) and each has precise indications. To date, from the hospital to the home setting, the monitoring and application of these respiratory support techniques have varied widely. In conclusion, the choice of respiratory support in this category of patients should be based on the technique that will optimize the comfort of the patient and improve the quality of their life. On the other hand, regarding monitoring, both telemedicine and ultrasound diagnostics help to satisfy the patient’s wish to spend the last period of his life in the home environment, to avoid inappropriately aggressive diagnostic interventions, and to reduce the high costs of hospitalized procedures in this category of patients.

## 1. Introduction

Over the past decade, palliative care (PC) has expanded to include chronic life-limiting respiratory diseases, including chronic obstructive pulmonary disease (COPD), severe refractory asthma, neuromuscular diseases with pulmonary exacerbations, neoplastic diseases with respiratory involvement, and acute respiratory failure in the elderly [1,2]. It is recommended that PC be initiated in the early stage of the diseases [3]. However, since there is no accepted method to predict prognosis or end stage of the diseases [4], that can allow to promote the early initiate of PC [5].

Dyspnea is the most common symptom, and other severe symptoms in patients with severe respiratory diseases, such as COPD, can have an impact on the patients’ ability to perform activities of daily living [6,7]. The experience of dyspnea is often associated with anxiety and panic, as well as concerns about death and dying [6,7,8].

The use of Non-invasive Respiratory Support (NRS), in particular after the SARS-CoV2 pandemic, is increasingly growing as a first-line treatment for patients with acute and acute-on-chronic respiratory failure who require hospitalization or home care support. NRS, known as Continuous Positive Airway Pressure (CPAP), Non-Invasive Positive Pressure Ventilation (NPPV), and High Flow Nasal Therapy (HFNT), are becoming the appropriate respiratory support for the management of severe dyspnea due to several physiological effects, i.e., better oxygenation and reducing the resistance load on the respiratory muscles, thus contributing to reducing the work of breathing [8]. In recent decades, a significant increase in the use of NPPV has been observed in elderly patients hospitalized with chronic respiratory disease. This suggests a major shift in the way healthcare professionals provide respiratory support in the palliative phase [9].

NPPV is the standard treatment for patients admitted to hospitals with COPD exacerbation and acute-on-chronic respiratory failure [9]. In this kind of patient, alleviation of dyspnea is considered crucial when NPPV is used as life support for patients who have decided to forgo invasive mechanical ventilation (IMV) or as a palliative measure [10].

HFNT is an alternative respiratory support that also could be offered to patients at the end of life to help relieve dyspnea from hypoxemia. NPPV is recommended in palliative care [11], although the discomfort and inability to eat or communicate may limit its use. Conversely, HFNT can be an alternative method to provide oxygen and respiratory support in a patient’s life-limiting chronic respiratory diseases. Several studies have reported the benefit of HFNT in relieving dyspnea in patients with advanced cancer and do-not-intubate patients with hypoxemic respiratory failure [12,13,14]. In addition, HFNT may be non-inferior to NPPV in alleviating dyspnea in patients with terminal cancer [15].

A growing body of evidence supports the use of NRS to improve patient comfort and quality of life. However, its role in palliative care is not well defined, and supporting evidence remains limited. The purpose of this narrative review is to examine recent evidence on the use of non-invasive respiratory support at the end of life to clarify who, how, and when it benefits. Specifically, we summarized the available data and address the different clinical scenarios in which NRS can be used during PC in specific populations, highlighting the different areas of application of non-invasive respiratory support, the characteristics of respiratory monitoring during NRS in PC, and the clinical settings in which NRS has been or may be applied in palliative care medicine.

## 2. Methods

The literature review was conducted by two subject matter experts with no predefined time limit in terms of years of search, on PubMed, using MeSH words. The types of papers included in the search were as follows: randomized controlled trials, systematic reviews, meta-analyses, guidelines, non-randomized controlled trials, narrative reviews, position papers, and experimental studies. Papers with a language different from English were excluded, as were conference proceedings, case reports, case series, and studies that included pediatric patients. The search terms included keywords related to the context such as ‘respiratory failure’, ‘palliative care’, ‘high flow nasal therapy’, non-invasive positive pressure ventilation, and end of life, as well as areas of interest such as ‘critical care’, and ‘home care’, were combined with ‘AND’ or ‘OR’.

## 3. Life-Limiting Respiratory Diseases with Palliative Care Needs

The treatment of patients with end-stage respiratory diseases is controversial.

Over the last decades, the intensive care survival rates among patients with severe respiratory conditions and elderly patients have increased. Intubation and invasive mechanical ventilation are a cornerstone in the treatment of acute respiratory failure (ARF). However, for a proportion of patients, the choice not to proceed with invasive mechanical ventilation depends on different factors such as patient’s wishes and patient’s clinical condition and comorbidities, which led the physician to believe that these measures cannot offer any therapeutic benefits.

In these conditions, palliative care plays a pivotal role. Key elements of palliative care include relief of pain and other distressing symptoms such as dyspnea, a team approach in addressing the needs of patients and their families, the enhancement of life, the mitigation of suffering and improvement of comfort, and the recognition of dying as a normal process bringing life to a natural end [16].

In this broad sense, palliative care should be considered early in the management of chronic disease and should not be restricted to patients admitted in hospice.

While palliative care has commonly been associated to terminal cancer patients, over the last decade, its role has become crucial as well in managing other life-limiting diseases, like chronic respiratory diseases (CRDs).

CRDs are among the 4 major human chronic diseases, accounting for an estimated 7.5 million deaths per year, approximately 14% of annual deaths worldwide [17].

Well-known CRDs include chronic obstructive pulmonary disease (COPD), lung cancer, infectious lung diseases, asthma, and interstitial lung diseases (ILDs). Most of the non-infectious conditions are related to smoking, indoor and outdoor air pollution, and occupational exposures [18]. Other conditions can lead to end-stage respiratory failure and dyspnea, such as solid and hematological malignancies and neuromuscular diseases.

COPD is a relevant cause of global morbidity and mortality, and its prevalence is expected to increase with global population growth and ageing [19].

The management for COPD is both pharmacological, based on inhaled bronchodilator and anti-inflammatory therapies, and non-pharmacological, based on long-term oxygen therapy and Non-invasive Positive Pressure Ventilation (NPPV) in the case of COPD exacerbation, which improves oxygenation and ventilation by reducing the work of breathing [20].

With the progression of the disease, COPD patients may develop persistent respiratory failure; thus, even when the treatment is optimized, they can experience a heavy symptom burden. Furthermore, many COPD patients also have multiple other comorbidities which have an impact on symptom burden and prognosis. In this context, palliative care can have a pivotal role with palliative therapies that can be delivered both in hospital and at home.

However, COPD patients are less likely than other patients with chronic illnesses to be referred to palliative care [21], because the disease has an unpredictable course and patients themselves have a limited understanding of their disease [6], and there is no clear definition of end-stage disease [4]. The European Respiratory Society task force recommended to start palliative care when an unmet need arises [22] and preferentially early, alongside disease-modifying therapies.

Lung cancer is the most frequent cause of cancer death, and its incidence is globally increasing. Tobacco smoking rate and stage of economic development influence its incidence and demographic distribution [23]. In advanced lung cancer, patients experience heavy physical symptoms ranging from pain and constitutional ailments of fatigue and anorexia to respiratory issues of dyspnea and cough [24]. Similarly, they experience physiological distress related to their diagnosis, prognosis, and quality of life. People with lung cancer commonly have exacerbations of congestive heart failure (CHF) and COPD as secondary precipitants of respiratory distress [25].

In patients with other solid or hematological malignancies, several conditions determine some forms of acute and chronic respiratory failure. Dyspnea is very common, affecting around 50–70% of patients with advanced cancer, and it can present also in mild hypoxemic or non-hypoxemic patients [26]. Pneumonia, pulmonary emboli, alveolar hemorrhage, cardiogenic pulmonary edema, lung injury related to chemotherapy are common causes of respiratory failure. Determining which of these patients will benefit from the Intensive Care Unit (ICU) is a crucial step to address them to invasive mechanical ventilation (IMV) or to palliative measures when there is a “do non intubate” order.

In the cancer trajectory, there is often a clear break between the phase of disease-modifying treatments and the end-stage palliative care [16]. However, there is increasing evidence that early palliative care from diagnosis improves outcomes for patients with lung cancer, thus palliative care and active anti-cancer treatments often run in parallel [27].

Chronic interstitial lung diseases (ILDs) are a heterogeneous group of fibrotic diseases including idiopathic pulmonary fibrosis (IPF), sarcoidosis, hypersensitivity pneumonitis, and connective tissue disease-associated ILD. Therapeutic tools are scarce in these diseases, and only a minority of patients meet eligibility criteria for lung transplantation [28]. Symptoms includes cough, fatigue, and increasing dyspnea.

Acute exacerbations of ILD are common in the disease course of ILD patients, often leading to hospital admission. An acute exacerbation can be unpredictable and idiopathic or triggered by intrinsic factors, like autoimmunity, and extrinsic factors, such as infections, aspiration, and drug toxicity. When an intensivist treats a deteriorating patient with acute exacerbation of ILD, the decision for IMV should be balanced with the prognosis and reversibility of the patient’s condition. Palliative care should be evaluated at patient’s admission, and a patient’s eligibility for lung transplantation plays a fundamental role in the decision algorithm.

Neuromuscular disorders (NMDs) include a group of genetic or acquired conditions where respiratory muscle weakness causes severe ventilatory restriction with a reduction in forced vital capacity, resulting in progressive respiratory failure. Mechanisms of dyspnea are complex in NMDs, as patients often describe their breathing as shallow or requiring undue work or effort, a consequence of increased neural drive despite normal respiratory system mechanics in most instances [29].

A lot of such illnesses still lack causative treatments. As weakness progresses, occurrence of hypoxic or hypercapnic respiratory failure requires ventilation support which must consider patient’s needs. Palliative measures should be considered early in these diseases, and for patients who refuse invasive mechanical ventilation and tracheostomy, the target of healthcare providers should be oriented to guarantee the “best” comfort avoiding undesirable measures.

## 4. Non-Invasive Respiratory Support (NRS) in Palliative Care: Which Options

In the clinical setting of end-stage chronic respiratory diseases, the predominant symptom which affects quality of life is dyspnea, associated to hypoxemic or hypercapnic respiratory failure.

The prevalence of severe dyspnea in patients who are terminally ill is about 70% with lung cancer and 90% with COPD [30].

**Figure 1:**
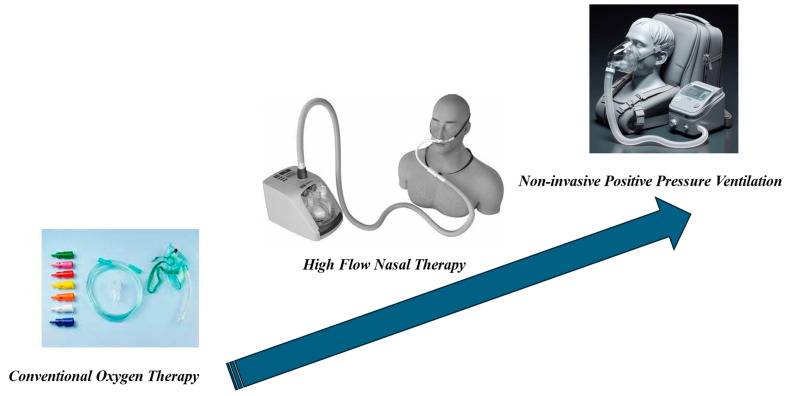
Non-invasive Respiratory Support (NRS).

Measures to alleviate dyspnea are pharmacological, such as opioids and anxiolytics, and non-pharmacological. These can include non-invasive measures such as conventional oxygen therapy, NPPV, and high-flow nasal therapy (HFNT) (Figure 1, Table 1 and Table 2).

### 4.1. Supplemental Oxygen

Conventional Oxygen Therapy (COT) is widely prescribed for relieving dyspnea in end-stage respiratory diseases, even in the absence of hypoxemia [31]. Over 70% of physicians caring for dyspneic palliative care patients report prescribing palliative oxygen, usually for refractory symptoms (65%) or at the patient’s request (30%) [32].

The oxygen delivery systems for domiciliary use include the conventional oxygen cylinders, oxygen concentrators, and liquid oxygen systems. Portable oxygen systems should be used in patients with an active lifestyle [33]. Oxygen can be delivered by simple face masks or nasal cannula, the latter being the preferred device as it allows eating and talking without major hindrance. Domiciliary long-term oxygen therapy (LTOT) is recommended in patients with chronic respiratory diseases and severe hypoxemia.

The relief of dyspnea in hypoxemic patients might occur by changes in chemoreceptor activation, the related reduction in respiratory drive and changes in breathing pattern, and/or stimulation of upper airway receptors by gas flow [34].

Despite potential benefits of oxygen use, only half of the patients use LTOT for the recommended daily use (>15 h/day). Patients’ compliance is affected by concerns about dependency and addiction, limitation of activity, mental confusion, and misunderstanding of the prescription and, in some parts of the world, inability to afford the cost of oxygen equipment. Furthermore, other barriers to optimal use of oxygen include concerns of physicians about risks of acute hypercapnia and lung damages due to oxidative stress.

The role of oxygen therapy to reduce severe dyspnea in patients without hypoxia is still controversial. In COPD patients with mild hypoxemia or exercise-induced oxygen desaturation alone, it has neither demonstrated to improve survival, exercise tolerance or symptoms nor to reduce hospital admission [35]. In a systematic review among mildly or non-hypoxemic patients with cancer [36], supplemental oxygen was not effective in reducing the sensation of dyspnea. In a cohort of 413 patients with refractory dyspnea, predominantly with cancer, when oxygen was prescribed on the basis of breathlessness alone, no improvement was shown [37].

An RCT [38] showed no additional symptomatic benefit of oxygen over room air delivered by nasal cannula for relieving refractory dyspnea related to life-limiting disease in patients with PaO_2_ > 55 mmHg. Dyspnea improved in both arms, irrespective of the administered gas and independently on any effect on PaO_2_.

In patients with advanced ILD, a systematic review showed no effects of oxygen therapy on dyspnea during exercise, although exercise capacity was increased [39].

In a multicentric RCT [40] in patients with fibrotic ILD without alteration of the oxygenation at rest and with isolated exertional hypoxia, ambulatory oxygen has been shown to be associated to health-related quality of life and could be an effective intervention in this patient group.

In prescribing LTOT, potential benefits should be carefully balanced with potential harms; patients’ adherence can be enhanced by patient education, regular follow-up and a home care program.

### 4.2. Non-Invasive Positive Pressure Ventilation (NPPV)

Non-invasive Positive Pressure Ventilation (NPPV) consists of positive pressure applied to the airways, and it can be delivered through different devices such as nasal, oronasal, and full-face masks and helmets [36], according to a patient’s anatomy and preferences.

In COPD patients with acute exacerbations and acute respiratory failure, NPPV is the standard treatment and is effective in reducing morbidity and mortality [41].

Domiciliary NPPV is often used in patients with COPD and hypercapnic respiratory failure [42].

Palliative NPPV in patients with limits on life support and treatments, such as patients with “do-not-intubate” orders and patients who receive only comfort measures, is still debated and controversial.

Clinicians who support the use of NPPV in these settings argue that there could be an improved chance of survival, an improvement in patient comfort near death, and that it provides additional time for patients to spend time with family and to fulfill end-of life tasks before death. Clinicians who are against the use of NPPV argue that it can delay the dying process, it can lead to unjustified use of health resources and can harm patients.

A recent review about use of NPPV in COPD patients with palliative care needs [43] found heterogeneous results about the perception of NPPV as a palliative measure by patients and healthcare practitioners. Many patients seemed to be satisfied with NPPV treatment as they perceived it as a way to keep them alive and alleviate symptoms. There were conflicting results as to whether NPPV improved dyspnea and health-related quality of life, even though some of the studies suggested that NPPV may reduce hospitalizations and COPD exacerbation and increase exercise capacity, and it allows patients to spend more time at home [44].

A study conducted in 18 cancer patients with life-support techniques limitation admitted for acute respiratory distress and receiving NPPV [45] concluded that NPPV appears to be an effective ventilation support in this group of patients.

In patients with hypercapnic respiratory failure, NPPV is more effective than in those with isolated hypoxemia, because it allows bilevel pressure ventilation to help remove carbon dioxide. Cuomo et al. [45] investigated the role of NPPV in 23 patients receiving palliative care and who were affected by severe hypoxic or hypercapnic ARF. After one hour, NPPV significantly improved PaO_2_/FiO_2_ and the Borg dyspnea score. NPPV also improved pH, but only in the subset of hypercapnic patients. Thirteen of these patients were successfully ventilated and discharged alive.

Another Randomized Control Trial (RCT) [46] suggests that NPPV is more effective compared with standard oxygen in reducing dyspnea and decreasing the doses of morphine needed in patients with end-stage cancer.

In patients with chest wall and NMDs, NPPV can help mitigate fatigue and dyspnea and make patients feel more in control of their disease [47]; it can also effectively reduce excessive daytime sleepiness in patients with nocturnal hypoventilation and sleep-related arousals [48].

Bourke et al. [49] found that in patients with amyotrophic lateral sclerosis, NPPV improved quality of life and survival, while in those with severe bulbar disfunction there was no benefit in terms of survival but still an improvement in quality of life, especially sleep-related symptoms.

### 4.3. High-Flow Nasal Therapy (HFNT)

HFNT is an oxygen therapy which can deliver warmed, humidified oxygen with a fraction of inspiratory oxygen (FiO_2_) up to 1, and a maximum flow of 60 L/min. HFNC has several physiological advantages: lung alveolar recruitment due to the generation of positive end-expiratory pressure (3–5 cmH_2_O), CO_2_ washout from the upper airway with a reduction in anatomic dead space [50], enhanced secretion clearance and expectoration [51], flow-dependent unloading of respiratory muscles based in part on the match between patient’s inspiratory needs, and the amount of the flow rate provided by the system.

HFNC has shown to be feasible and effective in patients with ARF.

Long-term HFNC therapy in COPD patients with chronic hypoxemic respiratory failure has been shown to reduce acute exacerbations, hospital admissions and the burden of symptoms and to improve patients’ sleep [52].

Compared to NPPV, HFNC is more comfortable, produces less skin injuries and allows eating and talking. In patients with hypoxemic respiratory failure, there is clinical evidence that HFNC may be superior to NPPV in terms of reduced 90-day mortality, with no differences in intubation rate [53].

In palliative care, however, potential advantages of HFNT over NPPV have not been proven.

In a retrospective study on 84 patients with a “do not intubate” order for hypoxemic respiratory failure associated with ILD, HFNT had a survival rate equivalent to that of NPPV but was better tolerated [13].

In another RCT [14], patients with advanced cancer and dyspnea were assigned to either HFNT or NPPV with Bilevel Positive Airway Pressure (BiPAP) modality for two hours; dyspnea was assessed by numerical rating scale and modified Borg scale. The results showed that both HFNT and NPPV reduced dyspnea and were safe, with no significant differences between the two interfaces.

In a real-life setting, the choice of the interface to manage dyspnea associated to end-stage respiratory diseases represents a challenge, as clear evidence is lacking. The choice among standard oxygen, NPPV and HFNT should rely on the presence of hypoxemia and/or hypercarbia and should be “patient-centered”, to respect his preferences and maximize his comfort at the end of life.

## 5. Indications for Respiratory Support in Chronic Respiratory Diseases in the Palliative Care Setting

In patients with advanced respiratory disease, non-invasive respiratory support strategies play a dual role in this multifaceted setting, taking the shape sometimes of a disease-modifying treatment, when addressed to the underlying causes of dyspnea, and in any case of a symptom-based intervention, aimed to reduce breathlessness and relieve suffering [54].

Table 1 and Table 2 depict the summarized clinical indications, recognized benefits and current concerns for each respiratory support previously described.

### 5.1. Conventional Oxygen Therapy (COT)

In patients with advanced respiratory disease, standard oxygen therapy is extensively prescribed for treatment of dyspnea, although its clinical effectiveness is established only in selected cases.

As regards COPD patients, current guidelines recommend long-term oxygen therapy (LTOT) only in the presence of hypoxemia, in virtue of a benefit on dyspnea relief and mortality [55]. In COPD patients without gas exchange impairment, on the contrary, there is no strong evidence to support the routine use of LTOT to alleviate dyspnea, but it should be used on an individualized basis [56]. There are no predictive factors to determine who will benefit, and a trial of oxygen could be carried out [57].

Similarly, in cancer patients mildly or not-hypoxemic, oxygen therapy does not provide symptomatic benefit in relieving dyspnea, as shown by a small meta-analysis by Uronis et al. [25]. Generalizing the issue, there is a lack of evidence supporting supplemental oxygen as a palliative end-life strategy, except in selected conditions [54].

Beyond guidelines and evidence, oxygen therapy is extensively prescribed and largely used in the palliative setting, and some patients reported a clinical benefit. Interestingly, a randomized clinical trial conducted by Abernathy and colleagues showed that oxygen therapy is not superior to room air in relieving refractory dyspnea in patients with life-limiting illness [37]. The main benefit of oxygen therapy, therefore, may rely on the mere air movement across the nasal cavities, a sort of “fan effect”. In addition, the psychological factor seems to play a crucial role in oxygen therapy patients’ request and clinicians’ prescription. A recent systematic review reported a triangular prospective (provided by patients, clinicians and caregivers) of using palliative oxygen for the symptomatic relief of breathlessness in people with advanced life-limiting illnesses. Most participants reported various clinical and phycological benefits (reduction of anxiety and breathlessness, improvement in sleep and quality of life), albeit some drawbacks were present [58].

As emerged, palliative oxygen therapy should be tailored to patients’ needs, preferences and clinical benefits.

### 5.2. Non-Invasive Positive Pressure Ventilation (NPPV)

The use of NPPV as a palliative measure is controversial and highly debated in the last thirty years [59]. According to some clinicians’ point of view, it is a feasible tool to relieve the subjective sensation of dyspnea, improve comfort in dying patients and even survival in those ones with a reversible component of dyspnea. It also provides additional time for patients to communicate with their family and fulfill end-of-life tasks before death, in line with a comprehensive palliative care strategy. Other clinicians, on the contrary, point out the harmful risk of prolonging the dying process, generating false hopes and unjustly allocating healthcare resources. Additionally, NPPV tolerability may be poor (due to claustrophobia, fogging of the interface, mucous dryness or skin ulcers), failing the key objective of palliative care. Thus, understanding goals of therapy, together with a careful evaluation of patient’s preferences and attitudes towards NIV, seems to be crucial and should be assured before proposing NPPV [60]. Accordingly, in 2007, a task force of the Society of Critical Care Medicine suggested a categorical approach to NPPV use in patients with acute respiratory failure, according to their advanced plan of care [9]:

- Category 1. Life support without preset limit
  In patients without preset limitations on advanced life-sustaining treatment, NPPV is a life support with the primary goals of assisting ventilation and oxygenation, relieving dyspnea and discomfort, and reducing the risk of intubation and mortality. NPPV use is aimed to restore health and intubation is indicated if NPPV fails.
- Category 2: Life support with preset limit—do not intubate (DNI order)
  In patients who decline endotracheal intubation and invasive mechanical ventilation, NPPV has the same goals of care as category 1, except for indication to intubation. In case of failure in improving gas exchange or unacceptable discomfort, that outweighed the potential benefits, NPPV should be discontinued, in favor of comfort measures only and other palliative symptom strategies.
- Category 3: Comfort measures only (CMO order)
  In patients with terminal malignancy or end-stage chronic disease refusing any form of life-prolonging therapy, NPPV is just a palliative measure, with the goal of maximizing comfort and relieving dyspnea. NPPV withdrawal is indicated in case of inability to provide further comfort and according to the patient’s desire. In line with that, there is no indication to deliver palliative NPPV in patients with severe sensorium alteration, as they do not experience distress.

A recent systematic review and meta-analysis summarized evidence in terms of survival, quality of life and tolerance of treatment when NPPV is delivered in patients with life-limited support (DNI or CMO orders) [60]. According to the twenty-seven studies on 2020 patients included, a significant proportion of patients with DNI orders and receiving NPPV for acute respiratory failure of any cause survived to hospital discharge (56%) and at 1 year (32%). Survival rate varied with the underlying condition (being higher in COPD or cardiogenic pulmonary edema than in pneumonia or cancer disease), without difference if NIV was delivered in an ICU or in a well-equipped hospital ward. NIV was generally well tolerated from the majority of patients and, among survivors, quality of life returned to baseline at 3 months.

Regarding NPPV use in patients with CMO orders, the former meta-analysis reported a high rate of acceptance (NPPV was stopped for intolerance or adverse effects in about 10% of patients only), although evidence is scarce [60]. Only one study, conducted by Nava et al., assessed the effect on dyspnea and opioid use. In their multicenter, randomized, feasibility study, NPPV resulted more effective than standard oxygen in reducing dyspnea, especially in patients with hypercapnia, and decreasing the total amount of morphine, with better cognitive function [46]. In light of this, the current clinical practice guideline of the European Respiratory Society suggests offering palliative NPPV to dyspneic patients in the setting of terminal cancer or other terminal conditions [10]. On the contrary, for patients with DNI orders, any recommendation was made, lacking randomized clinical trial [10].

Deepening the issue, NPPV’s role in life-limited illness may be differentiated according to etiology.

The available evidence strongly supports NPPV use in patients with end-stage COPD, given a well-recognized benefit on mortality and symptoms relief [61]. Moreover, in frail COPD subjects with a DNI order, NPPV was successful at reversing hypercapnic coma in severe acute-on-chronic respiratory failure [62]. Given the fluctuating course of COPD and the difficulty of predicting survival, it seems reasonable to offer an NPPV trial even in the case of supposed end-stage extreme acidotic respiratory failure. Recently, Steindal et al. [42] published a scoping review of 33 papers regarding NPPV use in COPD patients with palliative care needs. Although the included studies reported conflicting benefits and burdens, patients enrolled perceived NPPV more as a life-sustaining treatment than a palliative measure and expressed the desire to be included in the decision-making process regarding NPPV treatment [43].

In chest-wall and neuromuscular disease, NPPV seems to improve survival, quality of life and sleep disturbance, as well as palliate distressing symptoms. [63]. On the contrary, few studies support the use of NPPV in end-stage interstitial lung disease to date, and further research is warranted [64,65].

Finally, in oncologic patients with limited life support options, NPPV may be an option to relieve dyspnea, with a lower use of opioids, and also reverse respiratory failure if sustained by reversible factors, albeit with a significant hospital mortality rate [46,47]. To date, offering NPPV as palliative care to improve dyspnea has been recommended in patients with cancer [10].

In conclusion, NPPV may be feasible and effective as a palliative respiratory support, but a careful evaluation of a disease’s etiology, goals of therapy and patient’s preferences is required in order to assure clinical and ethical appropriateness.

### 5.3. High-Flow Nasal Therapy (HFNT)

The controversy linked to NPPV use and the uncertainty related to conventional oxygen therapy effectiveness have led to seek other respiratory strategies to support patients with end-stage diseases. Recently, High-Flow Nasal Therapy (HFNT) has been proposed in the palliative setting, as it is particularly appealing in virtue of its physiological and clinical effects, mainly the ability to relieve dyspnea with a high tolerability [66]. In a retrospective case series at Memorial Sloan-Kettering Cancer Center, the use of HFNT resulted safe and well tolerated, improving oxygenation and palliation [11]. Peters et al. reviewed the medical history of 50 patients with DNI directives and hypoxemia respiratory failure due to different underlying causes (fibrosis, pneumonia, COPD, cancer, hematologic malignancy, and congestive heart failure). HFNT was well tolerated, provided adequate oxygenation, and might be an alternative to NPPV for patients who decline intubation [13].

Recently, HFNT showed a therapeutic and palliative benefit, namely the same survival rate and a better tolerability compared to NPPV, in a retrospectively selected population of hypoxemic patients with interstitial lung disease and DNI orders [13]. Moreover, HFNT may have a role also as long-term domiciliary treatment alone or combined with NPPV in patients with heterogenous end-stage respiratory failure and discharged home [67].

Some prospective data come from a small phase II randomized trial of HFNT and NPPV in thirty cancer patients, showing a significant and similar relief of dyspnea with both strategies and suggesting the feasibility of and the need for large randomized controlled trials comparing HFNT and NPPV in this setting [14]. Currently, a large, prospective, observational study on patients with severe acute respiratory failure and DNI orders is taking place in 34 French ICUs [67]. The OXYPAL study is comparing three non-invasive strategies of oxygenation (HFNT alone, NPPV alternating with HFNT and NPPV alternating with standard oxygen) in terms of survival, symptom relief, tolerance and quality of life improvement.

In conclusion, HFNT seems to be a promising tool in palliative care, more effective than conventional oxygen therapy in relieving dyspnea and more tolerated than NPPV. Additional data are expected, and further research is needed to assess the potential role of this strategy in the context of palliative care.

## 6. Pharmacological Strategies for the Treatment of Symptoms That Require Non-Invasive Respiratory Support in the Palliative Care Setting

In the palliative care setting, managing symptoms that require non-invasive respiratory support involves a combination of pharmacological and non-pharmacological strategies aimed at improving patient comfort and quality of life.

Breathlessness is a predominant symptom that can be treated with a combination of drug therapies, such as opiates and benzodiazepines [68]. It is frequently linked to anxiety in a vicious cycle, and cognitive behavioral therapy techniques can be useful [69]. A non-productive cough may be alleviated by opiates [70]. Patients with chronic suppurative lung diseases, such as COPD, may have difficulty expectorating sputum and a particular fear of ‘choking to death’. Mucolytic drugs (e.g., hypertonic saline or carbocisteine) can be combined with sputum-clearing physiotherapy techniques.

The main drugs used for this purpose are (Table 3):

### 6.1. Opioids

In the context of non-invasive respiratory support for palliative care, it has been demonstrated that opioids are among the most widely used drugs. They have sedative and analgesic effects and can be the drugs of first choice when the primary objective is to reduce the perception of dyspnea with a consequent reduction in respiratory rate and an improvement in discomfort, thus increasing the acceptance of NRS [71]. Furthermore, they reduce pain control [72]. Morphine and fentanyl are widely used in hospital and domiciliary settings for palliative care context because of their efficacy in pain control and mitigation of psychological discomfort [73]. Dosing should be individualized, starting low and titrating up based on patient response and side effects. It has been demonstrated that when NRS is well tolerated, it can by itself alleviate the symptoms of these patients, thus allowing the possibility of reducing dosages and the related side effects. In patients with end-stage cancer, NRS is more effective compared with oxygen supplementation through Conventional Oxygen Therapy (COT) in reducing dyspnea and decreasing the doses of morphine needed [46].

The main side effects related to opioid infusion are hemodynamic alterations and the reduction in the caliber of the upper airways; moreover, opioids do not depress the respiratory drive but have an effect on respiratory timing. The introduction into clinical use of new synthetic opioids with limited adverse effects, particularly on the respiratory system, has offered an option for the analgesia sedation of critically ill patients. Among the opioid drugs on the market, remifentanil has a series of peculiar pharmacokinetic characteristics; its metabolism is not influenced by hepatic or renal dysfunctions, and the elimination half-life is less than 10 min, regardless of the duration of the infusion [73,74]. These characteristics make remifentanil easy to titrate and allow for the administration of opioids with fewer concerns about accumulation and unpredictable and/or delayed recovery. However, remifentanil, due to its pharmacokinetic properties, must be administered continuously; therefore, it finds a role exclusively in the hospital setting.

### 6.2. Benzodiazepines

Benzodiazepines are used to address anxiety and panic associated with breathlessness; they provide anxiolytic and sedative effects. Midazolam is commonly used, again with dosing tailored to the individual patient. However, it is necessary to consider some side effects of this category of drugs: benzodiazepines, in fact, can increase the incidence of delirium [75]. Moreover, midazolam has a significant impact on the circulation and respiration of patients, often causing hypotension and respiratory depression. Benzodiazepines can be used in the treatment of symptoms in palliative care patients with respiratory conditions, both in hospital and home settings.

### 6.3. Dexmedetomidine

Dexmedetomidine is a highly selective α2 agonist with sedative and hypnotic effects by acting on α2 receptors in the locus coeruleus and activating endogenous sleep-promoting pathways, allowing patients to maintain a natural sleep state [76]. A peculiar characteristic of dexmedetomidine is its ability to not cause respiratory depression and to allow the patient to be easily awakened. Furthermore, dexmedetomidine has a number of extremely advantageous characteristics: it has anxiolytic effects, reduces stress, provides good analgesia, inhibits the secretion of salivary glands, and has diuretic effects [77]. Dexmedetomidine also has a protective pulmonary effect, acting on the contractile mechanism of pulmonary vessels, on pulmonary vascular damage from ischemia–reperfusion, and on the release of inflammatory factors. Among the main side effects related to its infusion, bradycardia and hypotension have been described; however, by avoiding administering an initial bolus, these events, especially in more severe forms, are extremely rare [78]. Dexmedetomidine is a drug for exclusive hospital use.

### 6.4. Corticosteroids

Corticosteroids are commonly used for patients with airway obstruction, inflammation or tumor-related complications causing respiratory distress. They reduce inflammation and edema, improving airflow and reducing symptoms. Dexamethasone and prednisone are the first choice in this context. It has been demonstrated that the use of systemic corticosteroids for COPD exacerbations improves symptoms and lung function. Short courses of oral corticosteroids are in favor, leading to a decrease in pneumonia admissions and all-cause mortality [79]. Risk of adverse effects such as hyperglycemia is increased.

Systemic corticosteroids are commonly prescribed for palliation of dyspnea in patients with cancer. Hui et al. demonstrated that the use of high-dose dexamethasone did not improve dyspnea in patients with cancer more effectively than placebo and was associated with a higher frequency of adverse events [80].

The evidence supporting the use of systemic steroids as symptomatic drugs in palliation of dyspnea is weak and should not be prescribed for dyspnea where underlying severe airway or parenchymal lung involvement is not being managed.

### 6.5. Other Drugs

Among the various categories of drugs commonly used to reduce symptoms in patients with end-stage chronic pulmonary diseases (such as COPD and interstitial lung diseases), there are bronchodilators and anticholinergics. However, recommendations from guidelines are not always consistent with strong evidence. There is a shortage of high-quality studies focusing on patients with severe COPD.

## 7. Monitoring during Non-Invasive Respiratory Support in Palliative Care Setting

Non-invasive respiratory support (NRS) is a life-saving treatment that, unfortunately, may fail in some patients due to agitation or discomfort, primarily caused by intolerance to the interface (e.g., helmet, oro-nasal mask, and full-face mask) [80]. Additionally, suboptimal patient–ventilator interaction can contribute to this failure. Asynchrony between the patient and the ventilator occurs when there is a mismatch in breath delivery timing. This asynchrony is nearly inevitable due to mechanical and electrical delays within the complex patient–ventilator loop. Initial analyses on intubated patients mechanically ventilated in Pressure Support Ventilation (PSV) have shown that most asynchronous events occur during both inspiratory and expiratory phases. These events are caused by delayed or premature cycling of mechanical respiratory assistance, related to various ventilator settings such as inspiratory trigger, expiratory trigger, and pressurization ramp speed [81]. In hypoxemic patients, careful monitoring of the expired tidal volume is crucial. Tidal volumes above 9.5 mL/kg of ideal body weight indicate method failure [82]. Several studies have demonstrated that adjusting parameters regulating inspiratory and expiratory timing can improve patient–ventilator synchrony. Costa et al. found that using a faster pressurization rate (ramp) and a higher peak flow percentage for the flow trigger reduces inspiratory effort and asynchronies, particularly in patients with a high respiratory rate [81]. Even if patient–ventilator asynchrony is very common, studying this phenomenon in daily clinical practice is challenging [83]. Accurate assessment requires semi-invasive measurements of pleural pressure and/or respiratory muscle electromyogram [84]. Esophageal balloons, which provide a surrogate for pleural pressure, and respiratory muscle electromyograms have been employed to measure patient–ventilator interactions [85]. Assessing esophageal pressure, electrical activity of the diaphragm, electrical impedance tomography, and ultrasound of the lung and respiratory muscles can support physicians in managing acute respiratory failure, focusing on lung and respiratory muscle protection. However, these devices are not routinely used in patient care. Clinicians primarily rely on physical examinations and visual inspection of ventilator waveforms to assess patient–ventilator interaction.

Ventilator screens display Paw, volume, and flow waveforms in real time. While visual inspection of these waveforms generally correlates with esophageal-balloon readings, it is not error-free [86]. Limitations in detecting asynchronies from ventilator waveforms include:

- Even experienced ICU physicians may have difficulty reliably detecting asynchrony [83].
- Asynchronies can occur at any time, making continuous tracking impossible.
- Certain types of asynchronies, such as reverse triggering, are not easily detectable by inspecting pressure, flow, or volume waveforms alone.

The diaphragm ultrasound is a non-invasive, radiation-free imaging technique, making it suitable for repeated use without risks associated with other imaging modalities. Variation in diaphragm thickness during the respiratory cycle (thickening fraction, TFdi) correlates with respiratory pressure generation and electrical activity of the diaphragm (EAdi) [87]. TFdi can detect diaphragm weakness, with values below 30% during maximal inspiratory effort indicating significant weakness [87]. In mechanically ventilated patients, a progressive increase in diaphragm thickness over time may indicate excessive effort and under-assistance myotrauma. A TFdi of 15–30% during tidal ventilation is associated with stable diaphragm thickness and shorter ventilation duration [87]. Ultrasound is best suited for intermittent patient assessments rather than continuous monitoring. Diaphragm ultrasound is an increasingly valuable tool in the assessment and management of respiratory conditions, and its application in domiciliary (home) settings can offer significant benefits. Moreover, the diaphragm ultrasound in domiciliary settings offers a promising approach to enhance the management of chronic respiratory conditions, providing real-time, non-invasive assessment that can lead to better-tailored interventions and improved patient outcomes. With proper implementation, training, and integration into care plans, it can be a powerful tool in home-based respiratory care.

In the hospital setting, during NRS, particularly when sedative drugs are used, intensive monitoring is mandatory [88,89]. Key parameters to monitor include (Table 4):

- Heart rate and blood pressure (through ECG telemetry and invasive or non-invasive methods, with a maximum interval of 15 min).
- Respiratory dynamics, especially respiratory rate and use of accessory muscles.
- Ventilator waveforms (pressure and flow).
- Expired tidal volume in hypoxemic patients (when feasible).
- Gas exchange via arterial blood gas analysis.
- Level of consciousness using observational scales such as the RASS (Richmond Agitation-Sedation Scale) or the OAA/S (Observer’s Assessment of Alertness/Sedation Scale).
- Indices of NRS method failure, such as the ROX index and HACOR score [90].

Given the benefits of High-Flow Nasal Therapy (HFNT) in the acute setting for breathlessness and respiratory failure, along with its ease of use and patient comfort, domiciliary HFNT therapy is becoming an area of growing interest. Although HFNT devices are not portable due to the short tubing connecting the machine to the patient, the equipment is user-friendly for home use and is ideally employed during rest or at night. HFNT therapy, as a supportive treatment for breathlessness, is appealing and has advantages over conventional oxygen devices and non-invasive ventilation, which can cause claustrophobia and nasal pressure trauma. Additionally, studies on conventional domiciliary long-term oxygen therapy (LTOT) have indicated no benefit in relieving chronic breathlessness [25,37].

Domiciliary (home-based) respiratory monitoring is a critical aspect of managing chronic respiratory conditions. It involves the use of various tools and technologies to track respiratory health, detect early signs of deterioration, and adjust treatment plans accordingly. This approach helps improve patient outcomes, enhance quality of life, and reduce hospital admissions. Below is an overview of domiciliary respiratory monitoring.

### 7.1. Applications

Chronic Obstructive Pulmonary Disease (COPD): Monitoring exacerbations, oxygen levels, and ventilation patterns.

Asthma: Tracking peak expiratory flow rates and symptoms to manage and prevent attacks.

Sleep Apnea: Home sleep studies and continuous positive airway pressure (CPAP) compliance monitoring.

Neuromuscular Disorders: Assessing respiratory muscle strength and monitoring ventilation needs.

### 7.2. Tools and Technologies

Pulse Oximetry: Measures oxygen saturation (SpO_2_) and pulse rate. Useful for detecting hypoxemia.

Capnography: Monitors end-tidal carbon dioxide (ETCO_2_) to assess ventilation status.

Portable Spirometers: Measures lung function parameters like FEV1, FVC and PEF to track disease progression and response to treatment.

Peak Flow Meters: Used by asthma patients to monitor peak expiratory flow rates (PEFR).

Diaphragm Ultrasound: Assesses diaphragm function, useful in neuromuscular diseases and chronic respiratory failure.

Domiciliary respiratory monitoring is a valuable approach for managing chronic respiratory conditions, offering continuous oversight and enabling timely interventions. By leveraging a combination of advanced technologies, patient education, and seamless integration with healthcare systems, domiciliary monitoring can significantly improve patient outcomes and quality of life.

## 8. Quality of Life during Non-Invasive Respiratory Support in Palliative Care Setting

Quality of life (QoL) during NRS in a palliative care setting is a crucial aspect of patient management. Non-invasive respiratory support is commonly used to manage symptoms of respiratory distress in patients with chronic life-limiting respiratory disease [34].

Here are some key points on how NRS impacts QoL in palliative care:

### 8.1. Symptom Relief and Comfort

1. Breathlessness and Dyspnea:
  - NRS can significantly alleviate symptoms of breathlessness, a common and distressing symptom in palliative care patients.
  - Reduction in dyspnea can improve overall comfort and reduce anxiety associated with respiratory distress.
2. Sleep Quality:
  - Improved oxygenation and ventilation can enhance sleep quality by reducing nighttime awakenings and sleep fragmentation caused by apnea or hypopnea episodes [91].

### 8.2. Psychological Impact

1. Anxiety and Depression:
  - Effective symptom control can reduce anxiety and depressive symptoms associated with severe breathlessness and the fear of suffocation.
  - However, the presence of the mask and the noise from the machine can sometimes cause anxiety or claustrophobia in certain patients.
2. Sense of Control:
  - Patients often feel a greater sense of control over their breathing, which can enhance their overall sense of well-being and independence.

### 8.3. Social and Functional Aspects

1. Communication and Interaction:
  - NRS devices can interfere with verbal communication, making it difficult for patients to interact with family and caregivers.
  - This can be mitigated by scheduled breaks from the device or using communication aids.
2. Mobility and Daily Activities:
  - While NRS can improve energy levels and physical endurance by reducing the work of breathing, the equipment’s bulkiness and need for a power source can limit mobility.
  - Portable and more user-friendly NIRS devices are available and can help maintain a higher level of independence.

### 8.4. Comfort and Tolerance

1. Device Tolerance:
  - The fit and comfort of the mask, skin irritation, and dryness of the mouth or nasal passages are common issues that can affect QoL.
  - Proper mask fitting, humidification and regular skin care can alleviate these problems.
2. Compliance and Adaptation:
  - Patient education and support are crucial for improving compliance with NIRS.
  - A multidisciplinary approach involving respiratory therapists, palliative care specialists, and nurses can provide the necessary support and adjustments.

### 8.5. Ethical Considerations

1. Goals of Care:
  - Discussions around the goals of care and the patient’s wishes are essential. NRS should align with the patient’s overall care plan and end-of-life preferences.
  - In some cases, the use of NIRS may be more about symptom management than prolonging life, emphasizing comfort and QoL.
2. Decision-Making:
  - Informed decision-making involves discussing the potential benefits and burdens of NIRS with patients and their families.
  - Advanced directives and palliative care consultations can guide these decisions, ensuring that they align with the patient’s values and goals.

The impact of NRS on QoL in a palliative care setting is multifaceted, involving physical, psychological, social and ethical dimensions. By carefully considering and addressing these aspects, healthcare providers can optimize the use of NRS to enhance the QoL for patients in palliative care. Regular assessments and individualized care plans are essential to ensure that the benefits of NRS outweigh its challenges, always prioritizing the comfort and preferences of the patient.

## 9. Future Prospectives

To date, non-invasive respiratory support in the context of palliative therapies appears to be limited despite the growing need to ensure adequate support for patients who find themselves living with a chronic pathological condition for a prolonged period and for whom it is necessary to ensure an adequate quality of life and respiratory support.

Palliative care focuses on symptom relief by a multidisciplinary team and provides holistic care for patients and families as a unit. Although it is known that patients receiving palliative care for advanced chronic respiratory disease can benefit from specialist palliative care, many do not receive it unless they are referred for end-of-life care [6]. Referral to palliative care is influenced by the attitudes and knowledge of physicians about palliative care [92].

The optimal goal for the management of patients with life-threatening chronic respiratory illness would be to provide advanced palliative care (APC) for each individual patient [93]. APC models can improve outcomes for patients and families by using specific interventions and holistic approaches to care. APCs should provide a multidisciplinary approach (with palliative care specialists, physiotherapists, ethicists, pulmonologists) centered on the patient and the family–patient pair, as has been studied for COPD patients [93].

The management of respiratory support in patients with acute or acute-on-chronic respiratory failure in palliative care should move in this direction. The future goal of respiratory support in this category of palliative care patients should be to advance personalized medicine, integrate new technologies and improve interdisciplinary collaboration. In addition, increased awareness and education of healthcare providers and patients about the importance of respiratory support in palliative care will promote better outcomes and more humane care.

Indeed, in order to customize non-invasive respiratory support in patients who do not tolerate NPPV due to interface discomfort, feeding and communication limitation, several studies are examining the use of HFNT as non-invasive respiratory support in palliative care [94].

A future goal, in order to improve quality of life and patient comfort, could be to apply high flow in patients with life-limiting chronic respiratory disease not only in the hospital setting but also at home, managed by a multidisciplinary team.

Due to the complexity of advanced illness and its treatment, it can be difficult for palliative care patients to maintain their quality of life. Telemedicine interventions in the management of non-invasive respiratory support for patients with advanced and/or life-limiting chronic diseases could enable continuous monitoring by their healthcare providers, early detection of symptoms, and rapid response to respiratory management [95]. Castillo Padro and colleagues [95] have demonstrated that an intelligent remote patient monitoring system in clinical practice for patients in palliative care can improve access to health services and provide more information to professionals.

**Figure 2:**
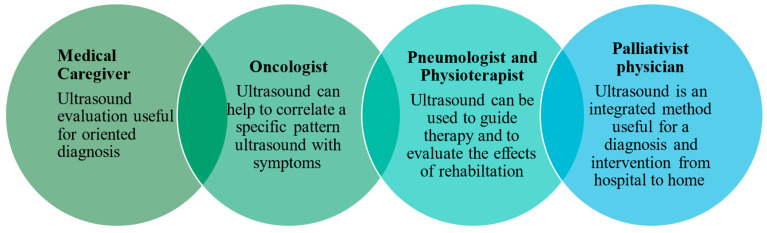
Role of ultrasound in palliative care.

Relying solely on the information available from the history and physical examination is often a reason for uncertainty among palliative care physicians in making treatment decisions during home visits, potentially leading to unnecessary hospital admissions or referral for cross-sectional imaging in radiology studies. A rational approach is essential to avoid diagnostic aggressiveness while providing the imaging information needed for optimal palliative care. The use of bedside handheld ultrasound has the potential to expand the diagnostic and therapeutic spectrum in cases of symptom exacerbation [96]. Lung ultrasound is the first and often the last diagnostic tool used and accepted by patients and clinicians. It also plays a key role in identifying palliative situations, providing follow-up care, identifying complications and safely implementing palliative interventions in hospital and home settings (Figure 2).

Another important goal in the management of non-invasive respiratory support in patients with advanced and/or life-limiting chronic diseases should be to mimic the environmental impact of such care while ensuring quality of care and ongoing patient and family support [97].

In addition, the increasing application of non-invasive respiratory support methods and non-invasive respiratory monitoring in the home setting could lead to a reduction in the infectious risk related to chronic life-limiting respiratory diseases [98].

To date, ongoing research into the effectiveness of different respiratory support interventions for patients with acute or acute-on-chronic respiratory failure in palliative care and their impact on quality of life will continue to inform best practice.

## 10. Limitations

This study has intrinsic limitations due to a narrative review, so no systematic revision of the literature was performed, and not all respiratory supports are considered. Furthermore, the research did not take into account the pediatric patient population that required a specific therapeutical approach.

## 11. Conclusions

In the management of respiratory support in patients with advanced and/or life-limiting chronic respiratory diseases, several studies have highlighted the importance of a multidisciplinary approach that integrates pharmacologic and non-pharmacologic therapies.

The choice of support depends on the patient’s clinical condition and the clinical care setting. High-flow oxygen therapy is increasingly being used to improve quality of life and patient comfort in the home environment.

Regarding the monitoring of the patient in respiratory support during palliative care being challenging, both telemedicine and ultrasound diagnostics help to fulfil the patient’s wish to spend the last period of life in their home environment, avoid inappropriately aggressive diagnostic interventions and reduce the high costs of procedures performed in hospital in this patient category.

A personalized approach targeting the needs and preferences of patients should be promoted, ensuring that each therapeutic strategy can improve the quality of life in the context of palliative care.

## Figures and Tables

**Table 1:** Special features of the setting and monitoring during the NRS.

Device	Indication	Setting	Monitoring
**Conventional Oxygen Therapy**	- COPD with hypoxemia (SpO_2_ < 88%) - Cancer patients with hypoxemia (SpO_2_ < 92%)	- Adjust flow (L/min) of oxygen delivered in order to achieve a value of SpO_2_ ≥ 92%.	- Peripheral Saturation (SpO_2_) - Respiratory Rate - Assessment of dyspnea
**High-Flow Nasal Therapy**	- COPD with dyspnea and hypoxemia (SpO_2_ < 88%) - elderly patients with hypoxemia (SpO_2_ < 92%) - patients with respiratory failure and DNI directives	- Choose the appropriate size of nasal cannula and circuit for the patient’s size. - Start with a minimum flow of 30 L/min. - Titrate the flow based on patient comfort and response (Changing air flows up to even 60 L/min) - Set the fraction of inspired oxygen (FiO_2_) to be delivered to achieve peripheral saturation ≥ 92%. - Adjustment of the humidification level in relation to patient comfort	- Peripheral Saturation (SpO_2_) - Respiratory Rate - Assessment of dyspnea - Assessment of comfort - Assessment of ROX index
**Non-invasive Positive Pressure Ventilation (NPPV)**	- COPD with dyspnea and hypoxemia (SpO_2_ < 88%) - elderly patients with hypoxemia (SpO_2_ < 92%) and dyspnea - patients with respiratory failure and DNI directives	- Choose the appropriate interface (oro-nasal mask, total full-face mask and Helmet) on patient comfort and clinical requirements. - Ensure a proper fit to minimize air leaks. - Set the fraction of inspired oxygen (FiO₂) to be delivered to achieve peripheral saturation ≥ 92%. - Set the inspiratory support so as to achieve a delivered tidal volume between 6 and 8 mL/kg - set PEEP on patient comfort, interface used, and clinical requirements. - Set the expiratory trigger based on patient’s respiratory mechanics characteristics.	- Hemodynamic parameters (blood pressure, heart rate) - Neurological status (Glasgow Coma Score, GCS) - Peripheral Saturation (SpO_2_) - Respiratory Rate - Assessment of dyspnea - Assessment of comfort - Assessment of HACOR score - Expiratory volume (Where it can be monitored) - Monitor the pressure and flow curves of the mechanical ventilator (evaluation of patient–ventilator synchrony)

**Abbreviations:** PEEP: Positive End Expiratory Pressure; HACOR: Heart rate, acidosis, consciousness, oxygenation, and respiratory rate; ROX index: Ratio of oxygen saturation; COPD: chronic obstructive respiratory disease; DNI: do not intubate.

**Table 2:** Pros and cons of the available non-respiratory support techniques in the palliative care setting.

Measure	Action	Pros	Cons
Conventional Oxygen Therapy (COT)	Relieve dyspnea even in absence of hypoxemia	- Possibility to adopt portable devices (low impact on quality of life) - Improvement in exercise capacity	- Lack of compliance in a high proportion of patients - Inability to afford the cost of oxygen equipment in low-income countries - Role of COT in reducing severe dyspnea in patients without hypoxia is still controversial
Non-invasive Positive Pressure Ventilation (NPPV)	Mitigation of static and dynamic hyperinflation, resistive load on the ventilatory muscles, thus reducing dyspnea and improving gas exchange	- Availability of different devices to better comply with individual necessities/preferences - More effective as compared to COT in reducing dyspnea and decreasing the doses of morphine needed in patients with end-stage cancer. - In patients with neuromuscular disorders, can help mitigate fatigue and reduce physical exhaustion	- Possible influence on life expectancy, thus raising concerns about the appropriateness of health resources’ use in this category of patients
High-Flow Nasal Therapy (HFNT)	Lung alveolar recruitment, CO_2_ washout (reduction of anatomic dead space), enhanced secretion clearance and expectoration	- In COPD patients with chronic hypoxemic respiratory failure, it has been shown to reduce acute exacerbations and hospital admissions - Low impact on patients’ quality of life and on daily activities	- Lack of robust data supporting the advantage of high-flow nasal therapy over NPPV in the palliative care setting

**Table 3:** Pharmacological alternatives to relieve dyspnea in the palliative care setting.

Drug Class	Compounds	Effect	Advantages	Disadvantages
Opioids	Morphine Fentalyn Remifentalin	Sedative and analgesic	Excellent control on pain Mitigation of psychological discomfort	Risk of hemodynamic alterations Need to individualize treatment schedule to avoid severe side effects
Benzodiazepines	Midazolam	Sedative and anxiolytic	Good efficacy in controlling anxiety and panic associated with breathlessness	Need to individualize treatment schedule to avoid severe side effects Significant impact on the circulation and respiration of patients (risk of hypotension and respiratory depression)
α2-agonists	Dexmedetomidine	Sedative and hypnotic	Reduces stress and provides good analgesia Additional protective effect on pulmonary vessels	Possible infusion-related side effects related (mainly bradycardia and hypotension) Exclusive hospital use
Corticosteroids	Dexamethasone Prednisone	Anti-inflammatory	Highly effective in improving airflow and reducing respiratory distress	Weak evidence supporting the use of systemic steroids as symptomatic drugs in palliation of dyspnea Risk of systemic metabolic side effects such as hyperglycemia

**Table 4:** A proposal of respiratory monitoring during non-invasive respiratory support in the palliative care setting.

Parameter	Monitoring Frequency	Monitoring Method	Normal Range/Findings
Respiratory Rate	Every 1–4 h	Visual observation, pulse oximetry	12–20 breaths per minute
Oxygen Saturation (SpO_2_)	Continuous	Pulse oximetry	≥95% on room air
Blood Pressure	Every 4–8 h	Automated blood pressure monitor, manual measurement	Systolic: 90–120 mmHg Diastolic: 60–80 mmHg
Heart Rate	Every 1–4 h	Automated monitor, manual palpation	60–100 beats per minute
Consciousness Level	Every 1–4 h	Glasgow Coma Scale (GCS), observation	GCS 15 (fully alert), appropriate for baseline mental status
Respiratory Pattern	Continuous	Visual observation	Regular, unlabored breathing without signs of distress
Respiratory Effort	Continuous	Visual observation	Even chest rise and fall, no use of accessory muscles
Skin Color and Temperature	Every 1–4 h	Observation	Pink and warm, consistent with baseline
Secretions	Every 1–4 h	Observation	Minimal or manageable, absence of excessive or copious
Pain Level	Every 4–8 h	Self-report, observation, pain scale assessment	Controlled and manageable, appropriate for baseline
Anxiety Level	Every 4–8 h	Self-report, observation, anxiety scale assessment	Controlled and manageable, appropriate for baseline

## Data Availability

Not applicable.
